# Assessing Biofungicides and Host Resistance against Rhizoctonia Large Patch in Zoysiagrass

**DOI:** 10.3390/pathogens13100864

**Published:** 2024-10-02

**Authors:** Bikash Ghimire, Rolando Orellana, Shukti R. Chowdhury, Christopher Brian Vermeer, Paige Patel, Paul Raymer, Susana Milla-Lewis, James W. Buck, Alfredo D. Martinez-Espinoza, Bochra A. Bahri

**Affiliations:** 1 Department of Plant Pathology, University of Georgia, Griffin Campus, Griffin, GA 30223, USA; bikash.ghimire@uga.edu (B.G.); cvermeer@uga.edu (C.B.V.); jwbuck@uga.edu (J.W.B.); amartine@uga.edu (A.D.M.-E.); 2 Institute of Plant Breeding, Genetics and Genomics, University of Georgia, Griffin Campus, Griffin, GA 30223, USA; praymer@uga.edu; 3 Georgia Center for Urban Agriculture, Extension Northwest District, University of Georgia, Griffin Campus, Griffin, GA 30223, USA; jrolando@uga.edu; 4 QAAFI, The University of Queensland, Brisbane, QLD 4072, Australia; shukti.chowdhury@sau.edu.bd; 5 Department of Soil & Crop Sciences, Texas A&M University, College Station, TX 77843, USA; pkpatel7303@gmail.com; 6 Department of Crop and Soil Sciences, University of Georgia, Griffin Campus, Griffin, GA 30223, USA; 7 Department of Crop and Soil Sciences, North Carolina State University, Raleigh, NC 27695, USA; srmilla@ncsu.edu

**Keywords:** *Rhizoctonia solani* AG2-2 LP, Rhizoctonia large patch, biofungicide, propiconazole, *Bacillus subtilis* QST713, zoysiagrass, host resistance, integrated disease management

## Abstract

Rhizoctonia large patch (*Rhizoctonia solani* AG2-2 LP) significantly reduces turfgrass quality, aesthetics, and playability. Synthetic fungicides are commonly used for managing this disease, but they present high costs, potential for fungicide resistance, and environmental concerns. We conducted in vitro assays to test the effectiveness of three biofungicides, seven synthetic fungicides, and ten combinations against *R. solani*. We then assessed seven spray programs that included *Bacillus subtilis* QST713 and propiconazole, either alone or tank-mixed, on zoysiagrass ‘El Toro’ in a growth chamber and in field trials. Biofungicide *B. subtilis* QST713 reduced pathogen growth by up to 100% in vitro. *B. subtilis* QST713 alone or combined with synthetic fungicides and/or in rotation was as effective as the standalone synthetic fungicide, reducing disease severity and AUDPC by 81 and 77% (growth chamber) and by 71 and 52% (field), respectively, while maintaining acceptable turfgrass quality. Additionally, we screened zoysiagrass genotypes and advanced breeding lines against three *R. solani* isolates in growth chamber studies. Five genotypes and two breeding lines demonstrated resistance to Rhizoctonia large patch across isolates, highlighting their potential for developing disease-resistant cultivars. Our findings suggest that integrating biofungicides, resistant cultivars with chemical controls offer sustainable and effective strategies for managing Rhizoctonia large patch

## 1. Introduction

Turfgrass, spanning 62 million acres, stands as the fourth largest crop in acreage in the United States, contributing significantly to the nation’s economy with an estimated value of USD 84 billion [1,2]. Maintaining disease-free turfgrass stands with high quality remains a persistent challenge. Among the various diseases threatening turfgrass, Rhizoctonia large patch (a.k.a. large patch) caused by *Rhizoctonia solani* AG2-2 LP, poses a significant challenge, compromising the aesthetic appeal and playability of sports fields. Large patch affects warm-season grasses including zoysiagrass, bermudagrass, centipedegrass, and St. Augustinegrass, while cool-season grasses such as bentgrass, bluegrass, fescues, and ryegrass are vulnerable to brown patch caused by *R. solani* AG-1 1A and AG2-2 IIIB [3,4]. Large patch can be a significant issue, especially during transitional seasons when warm-season turfgrass enters or exits dormancy [5,6]. Under favorable conditions at temperatures of 10 to 21 °C with prolonged moisture (≥48 h), *R. solani* can develop circular large patches of under 0.9 m up to 7.6 m, with matted areas of orange-bronze borders, consisting of water-soaked, reddish-brown to black lesions in leaf sheaths [7]. The severity of the disease is exacerbated by poor soil drainage, high compaction, thick thatch layers, low mowing heights, mechanical damage, and excessive nitrogen fertilization [7,8].

Management of large patch primarily requires an integrated approach that includes synthetic fungicides [4]. Some of the synthetic fungicides effective against large patch include methyl benzimidazole carbamates (MBCs) (FRAC group code 1), demethylase inhibitors (DMIs) (FRAC group code 3), succinate dehydrogenase inhibitors (SDHIs) (FRAC group code 7), and quinone outside inhibitors (QOIs) (FRAC group code 11) [4,8,9]. For example, azoxystrobin is a systemic strobilurin fungicide with preventive and curative properties to control a broad spectrum of more than 20 turfgrass diseases and it is highly efficacious against large patch [10]. Other highly efficacious fungicides include penthiopyrad and fluxapyroxad belonging to the succinate dehydrogenase inhibitors (SDHIs), registered recently, whereas boscalid (SDHI) has been on the market for over two decades. Fludioxonil (phenylpyrrole) is specific against basidiomycetes and is used against root and crown diseases of turfgrass such as large patch and take-all. Propiconazole is a widely used DMI fungicide for long-term and effective control of large patch, whereas mefentrifluconazol is one of the newest additions to this class [9,11]. However, research indicates that excess and repeated use of the same active ingredients can limit efficacy by giving rise to fungicide-resistant *R. solani* isolates in turfgrass [12] as reported for rice [13] and potato [14]. Therefore, it is imperative to identify and screen additional products that are efficacious against the disease, environmentally friendly, and safe for applicators and handlers.

Biological control agents (BCAs) offer promising avenues for mitigating soilborne pathogens like *R. solani* through various antifungal mechanisms including competition, antagonism, systemic resistance induction, and plant growth promotion [15,16]. For instance, *Bacillus amyloliquefaciens* SQR9 exhibits strong antagonistic activities against a broad spectrum of pathogens including *R. solani* through the production of lipopeptides (bacillomycin D, fengycin, surfactin) and a siderophore bacillibactin [15]. The whole genome analysis of the *B. subtilis* QST713 strain reveals fifteen gene clusters, nine of which are involved in the synthesis of surfactin, macrolactin, bacillaene, bacillomycin D, fengycin, difficidin, bacilysin, subtilin-like/ericin, and bacillibactin (siderophore) [17]. The pathogen dedicates at least 12% of its genome to the biosynthesis, regulation, and transport of antimicrobials [17]. *B. subtilis* QST713 and *B. amyloliquefaciens* SQR9 are used to develop broad-spectrum biofungicides Rhapsody and Stargus, respectively, which can reduce the risk of fungicide resistance given their multiple modes of action. Recently, Kim et al. [7] confirmed the inhibitory activity of *B. subtilis* SA-15 against *R. solani* in vitro arising from its antifungal compounds fengycin A and dehydroxyfengycin A. Additionally, BCAs like *Streptomyces neyagawaensis* and *Burkholderia vietnamiensi* have shown efficacy in suppressing large patch of zoysiagrass, while conferring enhanced tolerance to drought and temperature stresses [7,18]. Regalia, a plant extract from giant knotweed (*Reynoutria sachalinensis*, referred to as *R. sachalinensis* extr.) that activates the plant’s defense system to increase phenolics, phytoalexins, and antioxidants and lignification of plant cell walls, has also been used as a biostimulant for microbial control [19,20]. Given the prevalence of high disease pressure and the potential emergence of fungicide-resistant isolates in Georgia, there is a pressing need to explore alternative strategies for sustainable large patch management.

Host-plant resistance is an environmentally friendly and sustainable approach to large patch disease management in warm-season turfgrasses like zoysiagrass. However, limited efforts have been undertaken to identify sources of host plant resistance to large patch until recently. There are no highly resistant zoysiagrass cultivars available on the market to date [6], although over 50 improved cultivars have been developed since the introduction of three species of zoysiagrass [*Zoysia japonica* Steud., *Z. matrella* (L.) Merr., and *Z. pacifica* (Goudswaard) M. Hotta & S. Kuroki] into the US in 1892 [21,22]. Most of the breeding efforts in the past were focused on developing zoysiagrass cultivars with improved turfgrass quality characteristics, freezing, drought, and heat tolerance [23,24]. A four-phase collaborative effort among Texas A&M AgriLife Research, Kansas State University, and Purdue University developed a top 10 zoysiagrass hybrid with 15 to 40% lower large patch disease compared to Meyer [25]. As a result, a third-generation interspecific hybrid ‘DALZ 1701′ (hybrid ‘TAES 5723-47 × Meyer) was recently registered for the southern US and was found to have greater tolerance to large patch than Meyer [24]. Breeding for large patch resistance in zoysiagrass is currently underway at the University of Georgia and North Carolina State University (NCSU).

The objectives of the current study were to (1) develop alternative biofungicide-inclusive spray programs for efficient large patch disease management and (2) conduct host resistance screening in zoysiagrass genotypes and breeding lines against different *R. solani* isolates. Our long-term goals are to develop sustainable and effective management strategies for large patch disease, enhancing turfgrass health.

## 2. Materials and Methods

### 2.1. Inoculum Preparation

A total of three *Rhizoctonia solani* AG2-2 LP isolates were used in this study. Isolates Rs_Meyer2019 (GenBank Accession No. PQ009461) and Rs_SeaStar2022 (GenBank Accession no. PQ009462) were collected from the University of Georgia (UGA) Griffin Campus in 2019 and 2022 on zoysiagrass cv. ‘Meyer’ and seashore paspalum cv. ‘SeaStar’, respectively. Isolate LPZM2 (GenBank Accession no. PQ324967) was sampled from zoysiagrass cv. ‘Meyer’ in Raleigh, NC in 2011. Isolate Rs_Meyer2019 was employed in the biofungicide efficacy tests, while all three isolates were used in host resistance screening assays. The fungus was grown on potato dextrose agar (PDA) for seven days under a 12 h light cycle at room temperature for the in vitro biofungicide assay. For both growth chamber and field studies, the isolate was cultivated on a double sterilized oat/barley/wheat grain mixture, as described by Ghimire et al. [16]. Briefly, twenty agar plugs (7 mm in diameter) of the fungal isolates were added to 100 g of sterile grain in 250 mL Erlenmeyer flasks and cultured for 21 days at room temperature under a 12 h photoperiod. 

### 2.2. Bio- and Synthetic Fungicides

Three biofungicides comprising *B. subtilis* QST713 (Rhapsody™; FRAC Code BM 02), *B. amyloliquefaciens* F727 (Stargus™; FRAC Code BM 02), and *R. sachalinensis* extr. (Regalia™; FRAC Code P 05) were tested along with seven synthetic fungicides, comprising quinone outside inhibitor fungicide (FRAC Code 11) azoxystrobin (Heritage™), phenylpyrrole fungicide (FRAC Code 12) fludioxonil (Medallion™), succinate dehydrogenase inhibitor fungicides (FRAC Code 7) fluxapyroxad (Xzemplar™), penthiopyrad (Velista™), and boscalid (Emerald™), and demethylation inhibitor fungicides (FRAC Code 3) propiconazole (Banner Maxx™), and mefentrifluconazole (Maxtima™). All the fungicides were assessed against Rs_Meyer2019 in vitro, and *B. subtilis* QST713 and propiconazole were tested for their effectiveness against large patch in the growth chamber and field-based studies. Fungicides were formulated following the manufacturer’s instructions and applied on the same day they were prepared. Specifics on the formulations, application rates, and manufacturer details are provided in Table S1.

### 2.3. In Vitro Bio- and Synthetic Fungicide Efficacy Assays

In vitro experiments were conducted in two steps. First, the efficacy of the three biofungicides along with the seven synthetic fungicides was assessed in vitro against Rs_Meyer2019 isolate at their label rates (Tables S1 and S2) to select the best synthetic fungicide in controlling large patch. The experiment followed a completely randomized design using five petri dish replicates (100 × 15 mm) and was repeated once. The in vitro assay was based on the ‘poisoned food technique’ outlined by Grover and Moore [26]. The fungal plug was placed on the edge of the fungicide-amended PDA plate, and the longest mycelial radial growth was measured after four days of incubation at 25℃ under a 12 h photoperiod. The percent growth inhibition for each treatment compared to the non-fungicide amended control plate was calculated. In addition, ten combinations of biofungicides and synthetic fungicides that consisted of *B. subtilis* QST713, *R. sachalinensis* extr., and propiconazole at different tank mix ratios (25:75% 50:50%, and 75:25%) including the non-fungicide amended control were tested in vitro to identify the best treatment combination for further testing in the growth chamber and field studies (Table S2). The experiment was set up in a completely randomized design with five replications and was repeated once. The experimental setup, methods for data collection, and the parameters used were detailed earlier by Ghimire et al. [16].

### 2.4. Efficacy of Bio- and Synthetic Fungicide Spray Programs in Growth Chamber Experiments 

The efficacy of biofungicide *B. subtilis* QST713 was tested either alone or in combination with propiconazole in a tank mix in a growth chamber at the UGA Griffin Campus, Griffin, GA in 2023. Plugs (8.5 cm × 8.5 cm) of zoysiagrass cv. ‘El Toro’ were collected from the field and cultivated for three months in a greenhouse using Sungro professional growing mix (Sun Gro Horticulture, Agawam, MA, USA). The grass was maintained at a 5 cm height and fertilized with 0.7 gm/L water of Miracle-Gro^®^ Water Soluble All Purpose Plant Food (The Scotts Company LLC, Marysville, OH, USA) weekly as described by Ghimire et al. [16]. The experiment was set up in a repeated measure randomized complete block design with four replications. The turfgrass pots were artificially inoculated by placing four grains infested with Rs_Meyer2019 in the crown region and bagged in black plastic bags for 48 h in the greenhouse to maintain high relative humidity [16,27]. The inoculated pots were subsequently kept in a growth chamber for incubation for fourteen days (25/16 °C day/night; 12 h photoperiod) to develop large patch infection. The spray programs were initiated thereafter. Day 0 (hereafter referred to as the first day of the experiment) marked the start of the spray program and continued for six weeks (also termed as 42 days after the start of the experiment). 

In our study, the phrase ‘spray program’ is synonymous with ‘treatment’ since we applied more than one fungicide at different time intervals making a spray calendar. The seven spray programs included in our study are as follows: non-treated control with no fungicide application (T1), *B. subtilis* QST713 applied every 7 days (T2), *B. subtilis* QST713 applied every 14 days (T3), propiconazole applied every 28 days (T4), tank mix of 75% *B. subtilis* QST713 + 25% propiconazole applied every 28 days (T5), 75% *B. subtilis* QST713 + 25% propiconazole tank mix in rotation with 100% *B. subtilis* QST713 applied every 14 days (T6), and 100% *B. subtilis* QST713 in rotation with 75% *B. subtilis* QST713 + 25% propiconazole tank mix applied every 14 days (T7) (Table S3). Visual assessments of large patch severity were collected on each pot every seven days from 0 to 42 days using a modified Horsfall–Barratt scale of 1–11 [28]. The experiment was repeated once. Disease severity data were converted to a percentage scale, and the area under the disease progress curve (AUDPC) was computed using ARM statistical software (GDM Solutions, Inc., Brookings, SD) [29]. Analysis of variance was assessed for disease severity and AUDPC using R statistical software [30], and group means were compared using Tukey’s HSD test (*p* = 0.05). 

### 2.5. Efficacy of Bio- and Synthetic Fungicide Spray Programs in Field Experiments 

The seven spray programs tested in the growth chamber were assessed in the fall of 2022 and summer of 2023 at the UGA Griffin Campus in Griffin, GA (33°15′42.8″ N 84°17′02.1″ W) on zoysiagrass cv. ‘El Toro’ established in 2003 on clay loam soil (pH = 5.8). Turfgrass was managed according to the recommended management practices for golf course fairways in GA [31]. Turfgrass plots received irrigation every evening to ensure high humidity and were mowed to a height of 1.6 cm weekly. The experiment was arranged in a randomized complete block design in 1.5 m × 1.5 m plots with four replications. Turfgrass plots were artificially inoculated by placing 20 g of grain inoculum infected with Rs_Meyer2019 isolate per experimental plot two weeks before the start of fungicide spray programs. The seven spray programs were evaluated from 3 October to 14 November 2022 (fall season) and 8 May to 20 June 2023 (spring season) (Table S4). Two weeks after inoculation, fungicide spray programs were conducted using a hand-held CO_2_-pressured boom sprayer set at 30 psi, applying 81.5 mL of water per sq. m. through an XR TeeJet 8004VS nozzle. Large patch severity was visually estimated every seven days from 0 days (start of the spray program) to 42 days (end of the spray program) for each experimental plot using a modified Horsfall–Barratt rating scale (1–11) [28]. The severity scale was converted to percent disease severity using ARM statistical software, as described above [29]. Turfgrass quality was also visually assessed every week according to the National Turfgrass Evaluation Program (NTEP) guidelines using a 1–9 scale, where 1 is the poorest/dead, 6 or above is generally considered acceptable, and 9 is the best [32,33]. Data on percent disease severity, AUDPC, and turfgrass quality were subjected to analysis of variance in the R statistical program [30], and group means were separated using Tukey’s HSD test (*p* = 0.05). 

Based on results from the field experiments at the UGA Griffin Campus, two promising spray programs, *B. subtilis* QST713 applied every 7 days (T2) and a tank mix of 75% *B. subtilis* QST713 + 25% propiconazole applied every 28 days (T5), together with a non-treated control (T1) were further evaluated for their efficacy at Rivermont Golf Club in Johns Creek, Georgia (33°59′49.4″ N 84°15′52.6″ W) during the spring of 2023. This study aimed to encourage golf course superintendents to integrate biofungicides into their spray programs. The experimental site consisted of zoysiagrass cv. ‘Meyer’ established in 2006 and experiencing natural infections of large patch every year (Mark Hoban, Personal communication). The experiment was laid in a completely randomized design with four replications with an individual plot size of 1.5 m × 1.5 m. Evaluations of disease severity and turfgrass quality were conducted at three time points (0, 35, and 56 days) from 2 May to 27 June 2023 (Table S5), and the spray program was initiated at the first time point. The AUDPC was derived from disease severity. Spray techniques, fungicide doses, data collection, and statistical analysis followed similar to those described for field-based studies conducted at the UGA Griffin Campus.

### 2.6. Host Resistance Screening of Zoysiagrass against R. solani Isolates

#### 2.6.1. Screening Zoysiagrass Genotypes

A total of twenty-one zoysiagrass genotypes including plant introduction PI 231146 and PI 553020 were evaluated for their differential host resistance response against three isolates of *R. solani*, Rs_Meyer2019, Rs_SeaStar2022, and LPZM2 under growth chamber conditions at the UGA Griffin Campus in 2023. Accession PI 231146 is considered a potential source of resistance to large patch [34]. The experiment was arranged in a factorial randomized complete block design with three replicated pots (8.5 cm × 8.5 cm) using repeated measures. Inoculum preparation, inoculation, and growth chamber conditions followed the biofungicide efficacy experiment protocol, previously described. Disease severity was visually assessed weekly for six weeks starting 30 days post-inoculation, and the AUDPC was calculated. Cultivars were classified into three categories based on the AUDPC: resistant (R) with AUDPC < 350 (corresponding to disease severity < 10% at each time point), intermediate (I) with AUDPC = 350–1050 (corresponding to disease severity of 10–30% at each time point), and susceptible (S) with AUDPC > 1050 (corresponding to disease severity > 30% at each time point). Data analyses were carried out using the R version 4.3.3 software [30].

#### 2.6.2. Screening Zoysiagrass Breeding Lines

Five zoysiagrass advanced breeding lines developed by the UGA Turfgrass Breeding and Genetics Program at Griffin Campus using hybridization and subsequent field evaluations of progeny for turfgrass quality were evaluated alongside a moderately susceptible reference genotype ‘Zeon’ for resistance to *R. solani* isolate Rs_Meyer2019 at the UGA Griffin Campus in 2022. Inoculum preparation, inoculation, and growth chamber conditions followed the biofungicide efficacy experiment protocol, previously described. Disease severity was visually assessed at 16 and 36 days post-inoculation. The resistance level category followed as described above. The experiment was repeated once. Statistical analysis of the data was performed using the R version 4.3.3 software [30].

## 3. Results

### 3.1. Efficacy of Bio- and Synthetic Fungicide In Vitro

**Figure 1:**
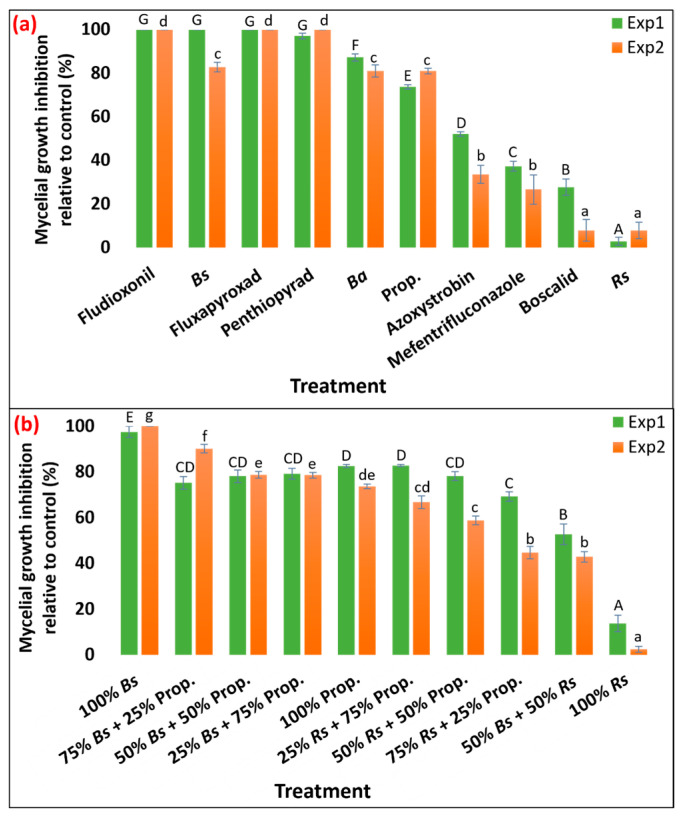
(**a**) Inhibition (%) of Rhizoctonia solani isolate Rs_Meyer2019 growth (%) in vitro under three biofungicides and seven synthetic fungicides and (**b**) ten combinations of two bio- and one synthetic fungicide at varying ratios compared to an untreated control. The green and orange bar represent first and second experiment, respectively. Average mycelial growth inhibition with identical letters on the bar chart shows no significant differences according to Tukey’s test (*p* = 0.05) and to separate analyses for Experiment 1 (Exp1) and Experiment 2 (Exp2). Error bars in the figure represent standard error of means. Prop. = Propiconazole; Bs = *Bacillus subtilis* QST713; Ba = *B. amyloliquefaciens* F727; Rs = *Reynoutria sachalinensis* extr.

The response of ten fungicide treatments against the mycelial growth of *R. solani* isolate Rs_Meyer2019 was evaluated across two independent experiments. Since the effects of ‘experiment’ (*p* = 7.27 × 10^−6^) and ‘treatment × experiment’ (*p* = 1.08 × 10^−7^) on mycelial growth inhibition were significant, the two experiments were analyzed separately (Table S6). Experiment 1 showed significantly higher mycelium inhibition compared to Experiment 2. In the first experiment, all treatments were significantly different from the control except *R. sachalinensis* extr. Biofungicide *B. subtilis* QST713, and fungicides fludioxonil, fluxapyroxad, and penthiopyrad almost entirely suppressed the growth of *R. solani* (97.1–100%) with no statistical difference among them (Figure 1a and Figure S1, Table S2). This group was followed by *B. amyloliquefaciens* F727 (87.3% mycelial growth inhibition) and propiconazole (73.7%), while biofungicide *R. sachalinensis* extr. exhibited the lowest mycelial growth inhibition (2.8%) compared to the control. Fungicides azoxystrobin, mefentrifluconazole, and boscalid showed moderate efficacy in controlling the mycelial growth of *R. solani* (27.7–52.0%) and were significantly different from each other and the rest of the fungicides. A similar result was observed in the second experiment for fungicides fludioxonil, fluxapyroxad, and penthiopyrad, with complete suppression of mycelial growth. Next to this group, biofungicides *B. subtilis* QST713 and *B. amyloliquefaciens* F727 and fungicide propiconazole yielded statistically similar mycelial inhibition (81.0–82.8%). Application of biofungicide *R. sachalinensis* extr. and the fungicide boscalid resulted in the lowest mycelial inhibition (7.8 and 7.9%, respectively) when compared to other treatments. The next group of fungicides, mefentrifluconazole and azoxystrobin, provided 26.6 and 33.6% inhibition of mycelial growth, respectively (Figure 1a and Figure S1, Table S2). Except for *R. sachalinensis* extr. and boscalid, all other treatments were significantly different from the control.

Additionally, the effectiveness of biofungicides *B. subtilis* QST713 and *R. sachalinensis* extr. and fungicide propiconazole was evaluated in vitro in three different combinations (1:3, 1:1, and 3:1). These combinations were compared against plates treated individually with each component and a non-treated control plate in two independent experiments. Since the effects of ‘experiment’ (*p* = 3.86 × 10^−10^) and ‘treatment × experiment’ (*p* = 7.62 × 10^−13^) on mycelial growth inhibition across ten fungicide treatments were significant, the two experiments were analyzed separately (Table S6). Experiment 1 displayed significantly higher mycelium inhibition compared to Experiment 2. In the first experiment, all treatments were significantly different from the control: 100% *B. subtilis* QST713-amended plate yielded nearly complete mycelial inhibition (97.5%), while a 100% *R. sachalinensis* extr. resulted in only 13.8% mycelial inhibition compared to the control. Six treatment combinations including 75% *B. subtilis* QST713 + 25% propiconazole (the treatment combination using the least amount of synthetic fungicide of all) followed the highest mycelial inhibition group with 75.2–82.8% suppression in mycelial growth (Figure 1b and Figure S2, Table S2). The second experiment illustrated a comparable trend with 100% *B. subtilis* QST713 and 100% *R. sachalinensis* extr.-amended plates resulting in the highest (100%) and the lowest (2.5%) mycelial growth inhibition compared to the control. The 75% *B. subtilis* QST713 + 25% propiconazole fungicide treatment held the second highest level of inhibition (90.1%), whereas the remaining seven treatments exhibited statistically distinct outcomes compared to the aforementioned treatments, showing 42.9–78.7% mycelial growth reduction relative to the control (Figure 1b and Figure S2, Table S2). All treatments were significantly different from the control except the plate amended with 100% *R. sachalinensis* extr.

### 3.2. Efficacy of Biofungicide-Inclusive Spray Program in Growth Chamber

The impact of seven spray programs across six distinct time points on disease severity and AUDPC was evaluated in two independent experiments conducted in the growth chamber. The effect of the ‘experiment’ was found to be statistically insignificant for disease severity across all time points (*p* = 0.413–0.896) except at 7 days and for AUDPC (*p* = 0.945). Consequently, data from both experiments were pooled for combined analysis (Table S7).

**Figure 2:**
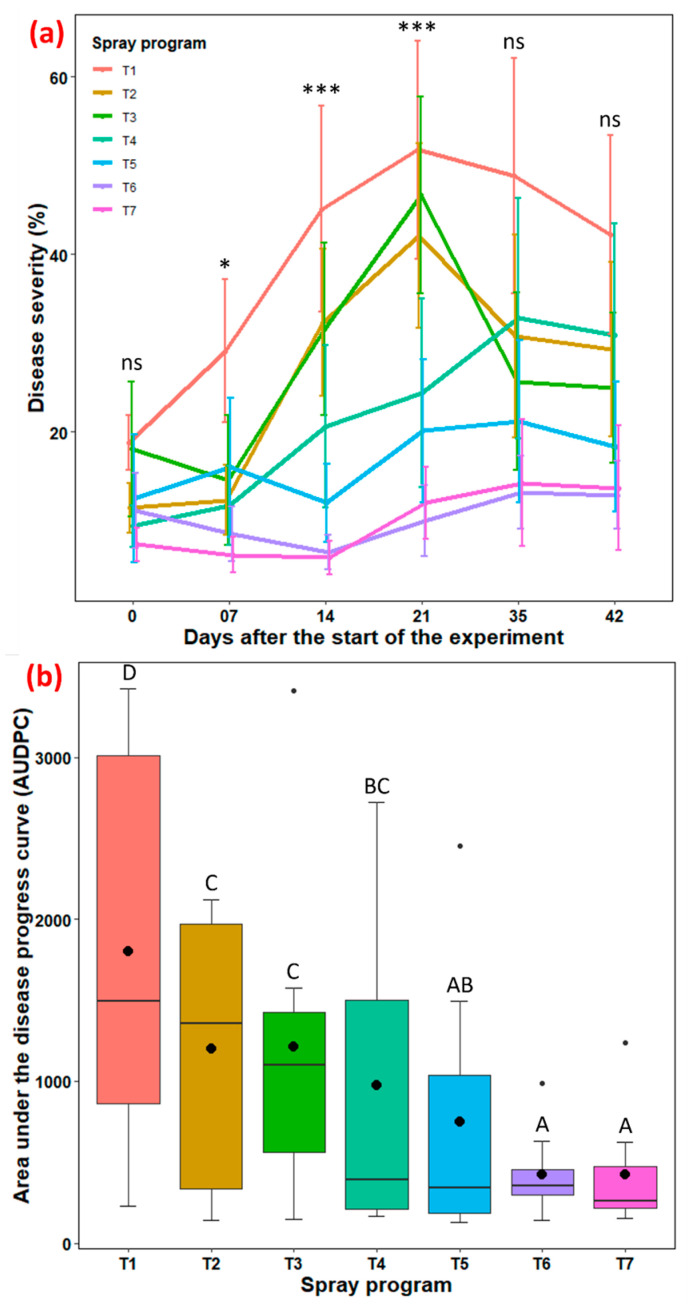
(**a**) Rhizoctonia large patch disease severity (%) for seven spray programs (T1–T7) across the six different time points (0–42 days) and (**b**) the area under the disease progress curve (AUDPC) in the growth chamber across two experiments. At 28 days, no data were collected. Tukey’s test showed a significant difference in average disease severity in panel (**a**) across seven spray programs at 7, 14, and 21 days (*p* < 0.05). ns represents non-significant at *p* < 0.05, * represent significant at *p* < 0.05, and *** represents significant at *p* < 0.001. Average AUDPC with the identical letters in the box plot in panel (**b**) are not significantly different according to Tukey’s test (*p* < 0.05). The black dot inside the boxplot in panel (**b**) is the mean value and the rrror bars in the figure represent standard error of means. T1: non-treated control; T2: *B. subtilis* QST713 applied every 7 days; T3: *B. subtilis* QST713 applied every 14 days; T4: propiconazole applied every 28 days; T5: tank mix of 75% *B. subtilis* QST713 + 25% propiconazole applied every 28 days; T6: 75% *B. subtilis* QST713 + 25% propiconazole tank mix in rotation with 100% *B. subtilis* QST713 applied every 14 days; and T7: 100% *B. subtilis* QST713 in rotation with 75% *B. subtilis* QST713 + 25% propiconazole tank mix applied every 14 days.

Disease severity: Significant differences (*p* = 0.046–< 2.0 × 10^−16^) in average disease severity among the seven spray programs were observed at 7, 14, and 21 days, while there were no significant differences in disease severity (*p* = 0.071–0.488) among spray programs at 0, 35, and 42 days after the start of the experiment (Figure 2a, Table S7). Disease progression accelerated rapidly for spray programs T2 and T3 until 21 days after which the disease started declining. Nevertheless, the remaining four spray programs (T4–T7) exhibited gradual and consistent progression until 35 days, after which severity levels plateaued for most of them. At 21 days after the start of the experiment (the last day a significant difference was observed), the T5, T6, and T7 spray programs, which integrate bio- and synthetic fungicides in a tank mix and rotation with biofungicide, exhibited significantly lower disease severities (10.0–20.1%), statistically differing from the non-treated control. This equates to a 61–81% reduction in large patch disease compared to the control (Table S3).

Area under the disease progress curve (AUDPC): Significant differences (*p* < 2.0 × 10^−16^) in AUDPC were detected among the seven spray programs. Spray programs 100% *B. subtilis* QST713 in rotation with 75% *B. subtilis* QST713 + 25% propiconazole tank mix applied every 14 days (T7), 75% *B. subtilis* QST713 + 25% propiconazole tank mix in rotation with 100% *B. subtilis* QST713 applied every 14 days (T6), and 75% *B. subtilis* QST713 + 25% propiconazole applied every 28 days (T5) demonstrated significantly lower AUDPC values (422, 424, and 751, respectively) (Figure 2b, Table S3). Both T6 and T7 achieved a slightly over 76% reduction in AUDPC, while T5 reported a 58% reduction compared to the control. In contrast, the non-treated control exhibited the highest AUDPC (1799), which was significantly higher than all other spray programs. The AUDPC for all other spray programs remained within the intermediate range (976–1215, equivalent to a 33–46% reduction compared to NTC) and were statistically similar to each other.

### 3.3. Efficacy of Biofungicide-Inclusive Spray Program in Field Experiments

#### 3.3.1. University of Georgia in Griffin, GA Experimental Site

The effect of seven spray programs on disease severity, AUDPC, and turfgrass quality were evaluated across seven different time points and two seasons in the field at the UGA Griffin Campus. Given the non-significant effects of ‘season’ and ‘season × spray program’ (*p* = 0.070–0.976; Table S8), data from fall and spring seasons were combined.

**Figure 3:**
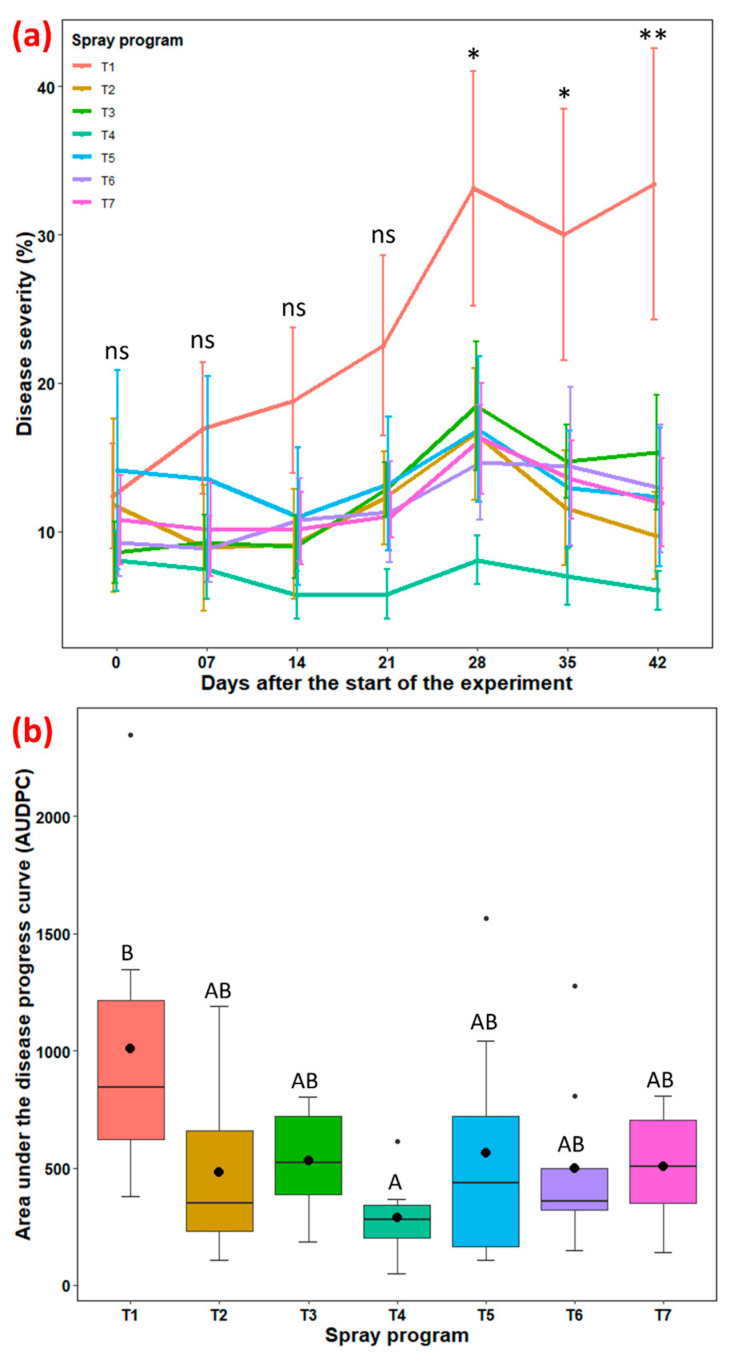
(**a**) Rhizoctonia large patch disease severity (%) for seven spray programs (T1–T7) across seven different time points (0–42 days) and (**b**) the area under the disease progress curve (AUDPC) in the field experiments across both fall 2022 and spring 2023 at the UGA Griffin Campus. Tukey’s test showed a significant difference in average disease severity in panel (**a**) across seven spray programs at 28, 35, and 42 days (*p* < 0.05). ns represents non-significant at *p* < 0.05, * represent significant at *p* < 0.05, and ** represents significant at *p* < 0.01. Average AUDPC with the identical letters in the box plot in panel (**b**) are not significantly different according to Tukey’s test (*p* < 0.05). The black dot inside the boxplot in panel (**b**) is the mean value and the error bars in the figure represent standard error of means. T1: non-treated control; T2: *B. subtilis* QST713 applied every 7 days; T3: *B. subtilis* QST713 applied every 14 days; T4: propiconazole applied every 28 days; T5: tank mix of 75% *B. subtilis* QST713 + 25% propiconazole applied every 28 days; T6: 75% *B. subtilis* QST713 + 25% propiconazole tank mix in rotation with 100% *B. subtilis* QST713 applied every 14 days; and T7: 100% *B. subtilis* QST713 in rotation with 75% *B. subtilis* QST713 + 25% propiconazole tank mix applied every 14 days.

Disease severity: A continuous increment in disease severity was observed in non-treated control plots with no significant difference with the other six spray programs until 21 days after the start of the experiment (*p* = 0.116–0.867; Figure 3a, Table S8). However, average disease severity was significantly different (*p* = 0.014–0.001) among the seven spray programs from 28 to 42 days. Average disease severities across six spray programs were almost at plateau before a slight increase at 28 days, followed by a subsequent decline. At 42 days, all six spray programs led to significantly lower disease severity, with percentages varying between 6.0% and 15.3% when compared to the non-treated control group (33.4%). The stand-alone use of propiconazole resulted in an 82% reduction in large patch severity and was comparable to the other five spray programs which reduced severity by 54.2 to 71.0% (Figure 3a, Table S4).

AUDPC: The overall effect of the spray program on AUDPC was not significant (*p* = 0.0585); however, Tukey’s test showed a significant mean separation between spray programs (Table S8). Non-treated control plots noted a higher AUDPC value (1010), while propiconazole applied every 28 days resulted in significantly lower AUDPC (286) among all (Figure 3b, Table S4). However, the other five spray programs showed no statistical differentiation among themselves or with the lower and higher groups, resulting in intermediate values for AUDPC (484–565). Overall, the stand-alone application of propiconazole led to a 71.7% reduction in AUDPC, while the other five biofungicide spray programs reduced AUDPC by 44.1–52.1%.

**Figure 4:**
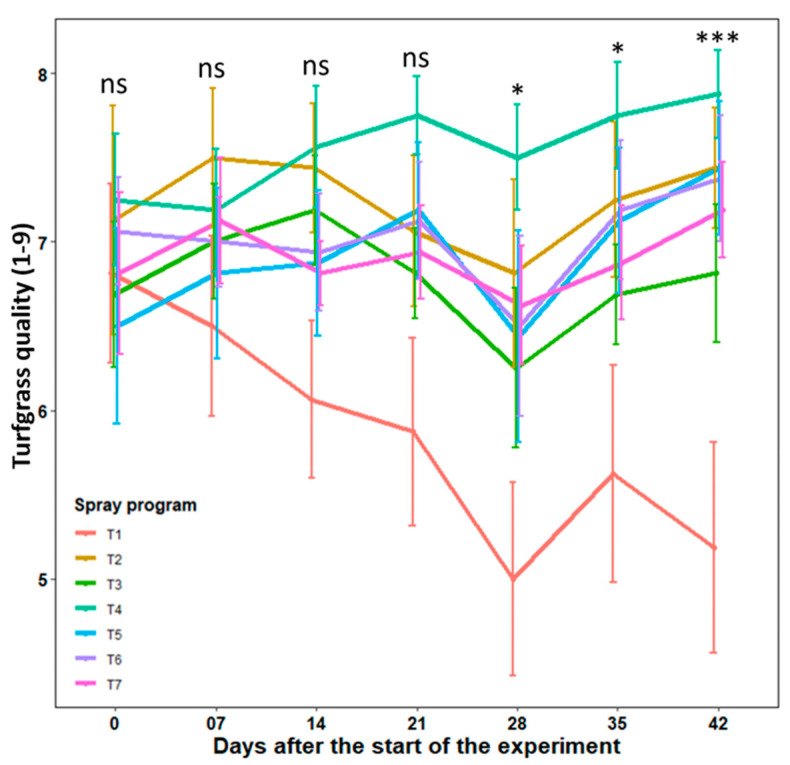
Turfgrass quality (1–9) obtained by applying seven spray programs (T1–T7) across the seven different time points (0–42 days) in the field experiments across fall 2022 and spring 2023 at the UGA Griffin Campus. Tukey’s test revealed a notable variation in average turfgrass quality across seven programs at 28, 35, and 42 days (*p* < 0.05). ns represents non-significant at *p* < 0.05, * represent significant at *p* < 0.05, and *** represents significant at *p* < 0.001. Error bars in the figure represent standard error of means. T1: non-treated control; T2: *B. subtilis* QST713 applied every 7 days; T3: *B. subtilis* QST713 applied every 14 days; T4: propiconazole applied every 28 days; T5: tank mix of 75% *B. subtilis* QST713 + 25% propiconazole applied every 28 days; T6: 75% *B. subtilis* QST713 + 25% propiconazole tank mix in rotation with 100% *B. subtilis* QST713 applied every 14 days; and T7: 100% *B. subtilis* QST713 in rotation with 75% *B. subtilis* QST713 + 25% propiconazole tank mix applied every 14 days.

Turfgrass quality: The quality of turfgrass continued to decline in non-treated control plots throughout the 42-day experimental period, except at 35 days (Figure 4, Table S4). Conversely, turfgrass quality continually improved for the spray program propiconazole applied every 28 days (T4). All remaining five spray programs sustained consistent recovery and improvement in quality beginning at 28 days when turfgrass quality reached its lowest level. No notable differences (*p* = 0.064–0.848) in turfgrass quality were observed among the seven spray programs until 21 days. However, after this period, a significant effect (*p* = 0.050–0.0003) of the six spray programs became evident when compared to the untreated control. At 42 days, the untreated control plots showed a notably reduced average turfgrass quality (5.2), which did not differ statistically from spray program *B. subtilis* QST713 applied every 14 days (6.8). The remaining five spray programs differed significantly from the control and resulted in higher and acceptable turfgrass quality (7.2–7.9), reaffirming the similar efficacy of biofungicide over stand-alone synthetic fungicide (Figure 4). Overall, turfgrass quality improved by 38.5–51.9% for five effective spray programs compared to the control.

#### 3.3.2. Rivermont Golf Club in Johns Creek, GA Experimental Site

Two spray programs, a stand-alone biofungicide *B. subtilis* QST713 (T2) and a tank mix comprising 75% *B. subtilis* QST713 + 25% propiconazole (T5) were selected for testing at Rivermont Golf Club in Johns Creek, GA, USA against the non-treated control plots (T1). The effect of the ‘spray program’ across three different time points for disease severity (*p* = 0.055–0.727), turfgrass quality (*p* = 0.214–0.693), and AUDPC (*p* = 0.171) were found non-significant (Table S9).

**Figure 5:**
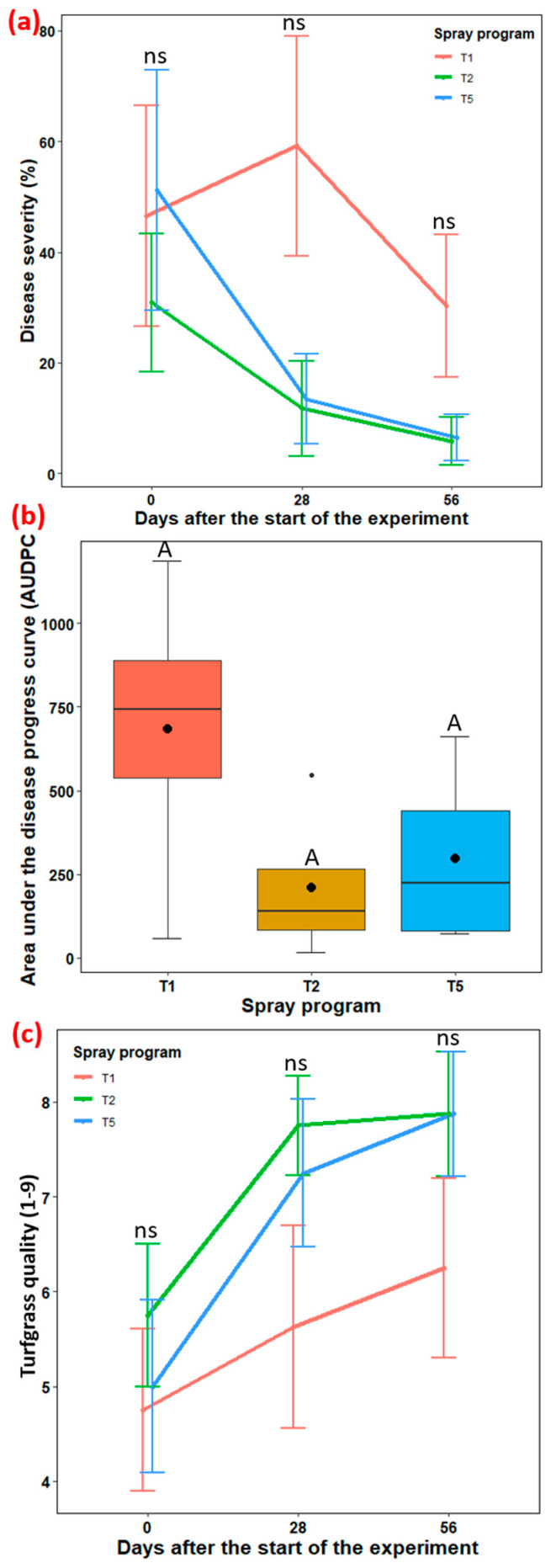
Efficacy of biofungicide spray program against Rhizoctonia large patch at Rivermont Golf Club in Johns Creek, GA during spring 2023. (**a**) Disease severity (%), (**b**) an area under the disease progress curve (AUDPC), and (**c**) turfgrass quality (1–9) obtained by applying three spray programs (T1, T2, and T5) across three different time points (0–58 days). Tukey’s test showed no significant differences in average disease severity (Panel (**a**) and turfgrass quality (Panel (**c**) across three spray programs at 28, 35, and 42 days (*p* > 0.05). ns represents non-significant at *p* < 0.05. Average AUDPC with the identical letters in the bar chart in Panel (**b**) are not significantly different according to Tukey’s test (*p* = 0.05). The black dot inside the boxplot in panel (**b**) is the mean value and the error bars in the figure represent standard error of means. T1: non-treated control; T2: *B. subtilis* QST713 applied every 7 days; and T5: tank mix of 75% *B. subtilis* QST713 + 25% propiconazole applied every 28 days.

Disease severity: Despite no significant difference being observed at all three time points, spray programs involving *B. subtilis* QST713 applied every 7 days (T2) and a tank mix of 75% *B. subtilis* QST713 + 25% propiconazole applied every 28 days (T5) noted 81 and 79% reduction in disease severity, respectively, compared to the non-treated control plot at 58 days after the start of the experiment (Figure 5a, Table S5). 

AUDPC: No significant differences in AUDPC were observed across the three spray programs despite values ranging from 210.5 to 682.9. Interestingly, AUDPC lowered by 69 and 57% with the T2 and T5 spray programs compared to the control, respectively (Figure 5b, Table S5). 

Turfgrass quality: Although non-significant, spray programs T2 and T5 exhibited highly acceptable turfgrass quality (7.9 for both) compared to the control (6.3) (Figure 5c, Table S5).

### 3.4. Host Resistance Screening of Zoysiagrass Genotypes

The effects of genotype (*p* < 2.0 × 10^−16^), isolate (*p* = 1.51 × 10^−3^), and their two-way interaction (*p* < 2.57 × 10^−14^) on AUDPC were significant (Table S10). Among the twenty-one zoysiagrass genotypes, Matrella exhibited a significantly lower AUDPC (114.8) when screened against the GA isolate Rs_Meyer2019 (Table 1). Genotype Geo showed the lowest AUDPC values (153.0) among all against the GA isolate Rs_SeaStar2022, whereas genotype Matrella (62.8) and plant introduction PI 231146 (60.0) recorded a lower value against the NC isolate LPZM2.

Three, five, and thirteen genotypes exhibited resistant, intermediate, and susceptible responses, respectively, when screened against the Rs_Meyer2019 isolate (Table 1). Matrella, PI 231146, and Pristine Flora were resistant to large patch disease. When tested against the Rs_SeaStar2022 isolate, one, eight, and twelve genotypes were grouped into resistant, intermediate, and susceptible categories, respectively, with genotype Geo showing resistance. Zoysiagrass genotypes Diamond, Matrella, and Pristine Flora and plant introduction PI 231146 exhibited resistance against the LPZM2 isolate, while the remaining eight and nine genotypes, respectively, displayed intermediate to susceptible response for large patch disease. Overall, *R. solani* isolate Rs_Meyer2019 resulted in significantly higher AUDPC (1628.4), while isolate LPZM2 noted the lowest AUDPC (1296.3). Isolate Rs_SeaStar2022 displayed an intermediate value (1433.4) and was not significantly different from other two isolates (Table 1). The disease progression over six weeks from 30 to 65 days post-inoculation is presented in Table S11. 

### 3.5. Host Resistance Screening of Zoysiagrass Breeding Lines

The disease severity of five zoysiagrass breeding lines as well as the reference cultivar Zeon was evaluated in two growth chamber experiments across two time points at the UGA Griffin Campus. Since the effect of the ‘experiment’ was found to be non-significant for disease severity at both time points (*p* = 0.178–0.579), data from the two experiments were analyzed together (Table S12). 

The effect of the ‘breeding line’ on disease severity was significant at both 16 (*p* = 6.40 × 10^−8^) and 36 days post-inoculation (*p* = 3.00 × 10^−5^) (Table S12). At 16 days, breeding lines GZZ LG3.20 (0), GZP 10.5.029.56 (1.2), HN 17-4 (1.6), and GZP 10.12.70.23 (3.5) exhibited significantly lower disease severity and were considered resistant to large patch (Table 2). At 36 days, breeding lines GZP 10.5.029.56 (5.9), HN 17-4 (9.8), and GZZ LG3.20 (11.7) and reference genotype Zeon (27.3) resulted in significantly lower disease severity, with the former two belonging to the resistant category while the latter two falling into the intermediate category.

## 4. Discussion

Management of Rhizoctonia large patch is largely dependent on chemical fungicides. Despite being considered a low-risk resistance pathogen, possibly due to the lack of spore production, fungicide resistance of *R. solani* has been reported across several regions of the world, including China [13,35], Tunisia [14], and the US [36]. A recent study by Ghimire et al. [16] revealed a surprisingly high level of fungicide resistance (66%) to propiconazole among *Clarireedia* spp. causing a dollar spot in Georgia turfgrass. This suggests a similar threat to the turfgrass industry from large patch pathogens, given the widespread use of propiconazole to control both diseases. The integration of biofungicides into fungicide spray programs and the use of disease-resistant cultivars present a promising multiprong approach to reduce reliance on synthetic fungicides [16]. Through our research, we were able to identify the best biofungicide-inclusive spray programs and large patch resistant zoysiagrass genotypes and breeding lines, which together could be a promising alternative to chemical fungicide for efficiently managing the disease. 

Our study demonstrated that biofungicide *B. subtilis* QST713 effectively reduced mycelial growth of *R. solani* by 83–100% in vitro. This finding is consistent with previous research. For instance, Ma et al. [37] reported that *Bacillus* spp. could reduce *R. solani* AG2-2 (IV) growth by up to 64% in vitro. Similarly, Kim et al. [7] observed the antagonistic effect of *B. subtilis* SA-15 inhibiting *R. solani* mycelial growth by 30–63%. A more recent study showed a 70–77% inhibition of *R. solani* strains causing brown or large patch due to *B. velezensis* GH1-13 [38]. We aimed to simulate a tank mix condition in our in vitro assay for which we tested three different ratios of *B. subtilis* QST713 in combination with the synthetic fungicide propiconazole. Our study revealed that a formulation consisting of 75% *B. subtilis* QST713 and 25% propiconazole achieved the highest fungal growth inhibition, reaching 90%. This combination, which used a minimal amount of synthetic fungicide and a substantial amount of biofungicide, showed promise for further testing. The high efficacy of both the standalone biofungicide *B. subtilis* QST713 and its combination with propiconazole in vitro provided sufficient evidence to formulate spray programs for in vivo testing. 

Our growth chamber study demonstrated a 61–81% reduction in large patch disease and a 58–77% reduction in AUDPC when combining a biofungicide with a synthetic fungicide in a tank mix and rotation. Previous research has advocated the benefits of tank-mixing fungicides or rotating high-risk fungicides with low-risk alternatives to delay or mitigate resistance development [39,40,41]. Consistent with our findings, Lee et al. [38] reported a 93% control efficacy of *B. velezensis* GH1-13 when tank-mixed with 50% azoxystrobin for controlling brown patch on creeping bentgrass in a greenhouse study, which was statistically comparable to 100% azoxystrobin (95%). Additionally, *Bacillus* spp. was found to reduce large patch disease by 28–43% in an in vivo growth chamber study conducted in South Korea [37].

Our field-based study revealed that *B. subtilis* QST713, whether applied alone or in combination with propiconazole, was as effective as propiconazole alone in reducing large patch severity by 54–71%. The highest level of disease control (71%) and the highest turfgrass quality (7.4) were achieved with weekly applications of *B. subtilis* QST713 (T2). Emerging evidence supports the efficacy of *Bacillus* spp. products in controlling large patch and brown patch diseases. A field trial by Kim et al. [7] confirmed a significant control efficacy of 51–92% against large patch with *B. subtilis* SA-15. This study further reported enhanced control efficacy (89–100%) when *B. subtilis* SA-15 was used in combination with half the standard dose of the synthetic fungicide pecycuron. Furthermore, a recent field-based study by Lee et al. [38] demonstrated higher control efficacy of *B. velezensis* strain GH1-13, either alone (77–89%) or in combination with a 50% azoxystrobin (81–82%), comparable to the efficacy of synthetic fungicide alone.

The top two spray programs, T2 and T5, identified from our two years of field-based studies at the UGA Griffin Campus, were further evaluated on an industry setting at Rivermont Golf Club in Johns Creek, GA. Although we did not observe any significant differences between the spray programs and the control plot, notably high control efficiency against large patch severity along with highly acceptable turfgrass quality was observed when *B. subtilis* QST713 was either applied alone weekly (81% and 7.9, respectively) or tank-mixed (75% *B. subtilis* QST713 + 25% propiconazole) and applied every 28 days (79% and 7.9, respectively). The lack of statistically significant differences may be attributed to the smaller sample size (*n* = 3) and replications (*n* = 4), which likely reduced the statistical power to detect true differences among the spray programs. Future studies should consider increasing replication size to address these limitations and improve the robustness of the findings. In addition, we relied on natural infection at Rivermont Golf Club compared to artificial inoculation at the UGA Griffin Campus. Therefore, differential disease pressure created at these two locations by possibly different pathogen strains and varying climatic conditions might be at play, resulting in altered efficacy of biological products as previously discussed in the literature [42,43].

Planting disease-resistant cultivars is another best alternative to chemical control, providing sustainable and environmentally friendly disease management practices to our golf courses, home lawns, and sports fields. Precise phenotyping and disease screening of current commercial genotypes and breeding pipelines provides critical information for breeding disease-resistant zoysiagrass [24,25]. Given the constant evolution of pathogens, which creates genetic diversity and impacts disease dynamics [44], it is crucial to explore novel resistance sources within existing germplasm pools. In this study, differential host resistance to three *R. solani* isolates was observed among 21 zoysiagrass genotypes in a growth chamber study. Georgia isolates Rs_Meyer2019 and Rs_SeaStar2022 showed contrasting infection levels on 9 out of 21 zoysiagrass genotypes. Interestingly, genotypes Matrella, Pristine Flora, and plant introduction PI 231146 which were resistant to Rs_Meyer2019 displayed intermediate to susceptible response to Rs_SeaStar2022. Similarly, genotype Geo was resistant to Rs_SeaStar2022 while susceptible to Rs_Meyer2019. This indicates that there is pathogen variability within Georgia. This variation could be attributed to the fact that the pathogens were isolated from different host types—zoysiagrass cv. ‘Meyer’ and seashore paspalum cv. ‘SeaStar’, which could influence their adaptations and preferences. 

Differential host resistance was also observed among *R. solani* isolates from North Carolina and Georgia, with contrasting infection levels on seven genotypes between Rs_Meyer2019 and LPZM2 and twelve genotypes between Rs_SeaStar2022 and LPZM2. There were four resistant genotypes against the North Carolina isolate LPZM2. Notably, genotypes Matrella, Pristine Flora, and plant introduction PI 231146 exhibited consistent resistance to both Rs_Meyer2019 and LPZM2. Four genotypes from our study were also previously screened by Zuleta et al. [27] against the same North Carolina isolate LPZM2 and the Florida isolate UF 0714, with plant introduction PI 231146 found to be stably resistant across all three isolates (Table S13). Shadow Turf displayed an intermediate reaction to the LPZM2 isolate in both studies. However, Cashmere and Cavalier exhibited intermediate reactions in Zuleta’s study [27], while they were resistant in the present study against the same isolate LPZM2. We speculate that this variability could be due to differences in the amount of grain inoculum and the incubation period between the two studies. In our studies, we used four grains to inoculate the plants in pots compared to 15 grains in Zuleta’s study [27], and data were collected 15 days after inoculation compared to 30 days after inoculation in our study. In contrast to our finding, Flor [6] did not identify any resistant zoysiagrass cultivars (severity < 20%) when inoculated with the Florida isolate UF 0714 in a plant growth room, among a collection of 12 commercial cultivars, 10 of which were also included in our study. While Empire exhibited the lowest disease severity for the Florida isolate in Flor’s study [6], it was found to be intermediate to susceptible for three isolates in our research. Consistent with our study, recent data from the 2022 and 2023 NTEP field trials showed intermediate to higher disease pressure on Empire, Emerald, Zeon, and Meyer in Jay, FL, except for Meyer in 2022 [45,46]. In contrast, minimal to no disease was observed in these cultivars in 2020 in Dallas, TX [47]. In another study at NCSU, genotype Zeon was found to be highly resistant, while Meyer was susceptible [34]. Obasa et al. [48] reported intermediate disease incidence (42–50%) on Meyer in 2009–2010 growth chamber studies against the Kansas isolate. Different geographic locations with varying climatic conditions, fungicide use patterns, and pathogen strains were possibly responsible for this differential cultivar response. 

The five resistant genotypes (Diamond, Geo, Matrella, PI 231146, and Pristine Flora) identified in our study hold considerable promise. These genotypes could either be directly recommended for use in different turfgrass industry settings or could be utilized for identification of resistance genes that can be incorporated in breeding pipelines. Additionally, the two consistently resistant breeding lines, GZP 10.5.029.56 and HN 17-4, which maintained resistance throughout the 36-day post-inoculation period, represent valuable genetic resources that could further be utilized for making crosses to develop disease-resistant cultivars.

A strategic approach is essential to address the knowledge gap on *R. solani* strain diversity, which can lead to the development of fungicide-resistant populations. Continuous surveillance and comprehensive sampling of *R. solani* isolates across diverse geographical regions are vital to understanding genetic diversity and establishing baseline fungicide sensitivity, particularly as pathogen populations are constantly evolving in response to changing climatic conditions and varying fungicide pressures [9,39]. Additionally, rigorous screening and pathogenicity testing among warm-season turfgrass cultivars, including zoysiagrass, are imperative for assessing the virulence of emerging pathogen strains and their adaptability to different host types. This multifaceted strategy will provide researchers and turfgrass managers with critical insights, enabling more effective disease management practices. Overall, integrating biological fungicides with resistant cultivars potentially obviates the reliance on conventional chemical treatments, which otherwise lead to reduced fungicide sensitivity, ultimately achieving sustainable large patch disease management and significantly enhancing turfgrass health and quality.

## Figures and Tables

**Table 1:** Area under the disease progress curve (AUDPC) for Rhizoctonia large patch on 21 zoysiagrass genotypes, including plant introduction accessions PI 231146 and PI 553020, inoculated with three *Rhizoctonia solani* isolates and assessed weekly after 30 days post-inoculation across six weeks in a growth chamber experiment at the University of Georgia (UGA) Griffin Campus in 2023. Cultivars were classified into three categories: resistant (R) with AUDPC < 350 (presented in bold font), intermediate (I) with AUDPC = 350–1050, and susceptible (S) with AUDPC > 1050, as mentioned in the table. Average AUDPC values with the identical letters across all three isolates and 24 cultivars are not significantly different according to Tukey’s test (*p* = 0.05).

Genotypes	*Rhizoctonia solani* Isolates						Mean	
	Rs_Meyer2019		Rs_SeaStar2022		LPZM2			
Cashmere	1706.2 ± 366.5 ^abcdefghijkl^	S	900.5 ± 136.4 ^abcde^	I	698.1 ± 412.8 ^abcde^	I	1101.6 ± 6.3 ^BCD^	S
Cavalier	1308.8 ± 556.6 ^abcdefgh^	S	1708.9 ± 160.0 ^abcdefghijkl^	S	700.8 ± 185.0 ^abcde^	I	1239.5 ± 6.6 ^CD^	S
Diamond	998.0 ± 462.2 ^abcde^	I	1185.7 ± 546.7 ^abcdefg^	S	**183 ± 29.0 ^ab^**	**R**	788.9 ± 7.5 ^ABCD^	I
El Toro	2794.6 ± 88.9 ^ghijklm^	S	3018.8 ± 207.5 ^hijklm^	S	1637.9 ± 158.1 ^abcdefghijk^	S	2483.7 ± 6.3 ^FG^	S
Emerald	3204.7 ± 115.7 ^jklm^	S	3456.3 ± 11.9 ^m^	S	2850.2 ± 246.5 ^ghijklm^	S	3170.4 ± 3.7 ^G^	S
Empire	1730 ± 384.7 ^abcdefghijklm^	S	1184.9 ± 217.2 ^abcdefg^	S	607.8 ± 19.1 ^abcde^	I	1174.2 ± 5.4 ^CD^	S
Geo	3322.3 ± 38.3 ^klm^	S	**153.0 ± 27.7 ^ab^**	**R**	1009.0 ± 306.1 ^abcdef^	I	1494.7 ± 13.6 ^CDE^	S
Innovation	3363.3 ± 64.5 ^klm^	S	1186.7 ± 281.1 ^abcdefg^	S	3177.3 ± 142.3 ^jklm^	S	2575.8 ± 10 ^FG^	S
JaMur	1977.9 ± 251.0 ^cdefghijklm^	S	430.1 ± 40.6 ^abcd^	I	1538.5 ± 471.4 ^abcdefghij^	S	1315.5 ± 7.3 ^CD^	S
Matrella	**114.8 ± 37.0 ^a^**	**R**	397.3 ± 244.5 ^abc^	I	**62.8 ± 39.4 ^a^**	**R**	**191.6 ± 2.4 ^A^**	**R**
Meyer	692.6 ± 100.7 ^abcde^	I	1121.0 ± 339.6 ^abcdefg^	S	798.4 ± 142.2 ^abcde^	I	870.7 ± 3.6 ^ABCD^	I
Palisades	1222.2 ± 429.5 ^abcdefg^	S	2748.1 ± 222.6 ^fghijklm^	S	721.8 ± 180.8 ^abcde^	I	1564 ± 9.5 ^DE^	S
PI 231146	**261.5 ± 171.0 ^abc^**	**R**	382.7 ± 107.7 ^abc^	I	**60.0 ± 10.9 ^a^**	**R**	**234.7 ± 2.3 ^AB^**	**R**
PI 553020	1563.1 ± 228.2 ^abcdefghij^	S	3128.1 ± 80.1 ^ijklm^	S	1875.8 ± 612.4 ^bcdefghijklm^	S	2189 ± 8.3 ^EF^	S
Pristine Flora	**257.0 ± 42.5 ^abc^**	**R**	1797.4 ± 267.3 ^abcdefghijklm^	S	**144.8 ± 69.0 ^ab^**	**R**	733 ± 7.9 ^ABCD^	I
Rollmaster	747.3 ± 414.4 ^abcde^	I	359.9 ± 182.4 ^abc^	I	806.5 ± 366.8 ^abcde^	I	637.9 ± 5.3 ^ABC^	I
Shadow Turf	1169.3 ± 581.1 ^abcdefg^	S	994.3 ± 481.8 ^abcde^	I	1392.6 ± 399.7 ^abcdefghi^	S	1185.4 ± 7 ^CD^	S
Trinity (formerly L1F)	3148.2 ± 73.5 ^jklm^	S	2203.0 ± 112.2 ^efghijklm^	S	3401.6 ± 0.02 ^lm^	S	2917.6 ± 5.4 ^FG^	S
Zenith	2993.2 ± 159.1 ^hijklm^	S	2154.7 ± 320.1 ^defghijklm^	S	3317.8 ± 113.2 ^klm^	S	2821.9 ± 5.8 ^FG^	S
Zeon	951.5 ± 422.0 ^abcde^	I	712.6 ± 335.6 ^abcde^	I	668.0 ± 365.4 ^abcde^	I	777.4 ± 5.4 ^ABCD^	I
Zorro	668.9 ± 342.2 ^abcde^	I	876.7 ± 480.2 ^abcde^	I	1570.4 ± 643.8 ^abcdefghij^	S	1038.7 ± 7.8 ^ABCD^	I
Mean	1628.4 ± 146.7 ^B^		1433.4 ± 133.5 ^AB^		1296.3 ± 144.0 ^A^		1452.3	

**Table 2:** Disease severity (%) for Rhizoctonia large patch on five zoysiagrass breeding lines, inoculated with *Rhizoctonia solani* isolate Rs_Meyer2019 and assessed at two time points (16 and 36 days post-inoculation) across two growth chamber experiments at the UGA Griffin Campus in 2022. Genotype Zeon was included as a moderately susceptible reference. Breeding lines were classified into three categories: resistant (R < 10% disease severity) (presented in bold font), intermediate (I = 10–30% disease severity), and susceptible (S > 30% disease severity), as mentioned in the table. Average disease severity values with the identical letters within each date are not significantly different according to Tukey’s test (*p* = 0.05).

Breeding Lines	Day 16		Day 36	
GZP 09.03.12	16.4 ± 4.8 ^b^	I	53.9 ± 15.7 ^bc^	S
GZP 10.12.70.23	**3.5 ± 0.5 ^a^**	**R**	74.4 ± 9.8 ^c^	S
GZP 10.5.029.56	**1.2 ± 0.5 ^a^**	**R**	**5.9 ± 1.2 ^a^**	**R**
GZZ LG3.20	**0.0 ± 0.0 ^a^**	**R**	11.7 ± 3.3 ^a^	I
HN 17-4	**1.6 ± 0.8 ^a^**	**R**	**9.8 ± 5.7 ^a^**	**R**
Zeon	30.7 ± 7.8 ^c^	S	27.3 ± 6.5 ^ab^	I

## Data Availability

Data are available in a publicly available repository.

## Supplementary Materials

The following supporting information can be downloaded at: https://www.mdpi.com/article/10.3390/pathogens13100864/s1. Figure S1: In vitro growth of *Rhizoctonia solani* isolate RS_Meyer2019 in fungicide-amended potato dextrose agar (PDA) plate after four days of incubation; (a) control with no fungicide, (b) *Bacillus subtilis* QST713 (Rhapsody), (c) *B. amyloliquefaciens* F727 (Stargus), (d) *Reynoutria sachalinensis* extr. (Regalia), (e) azoxystrobin (Heritage), (f) boscalid (Emerald), (g) fludioxonil (Medallion), (h) fluxapyroxad (Xzemplar), (i) mefentrifluconazole (Maxtima), (j) penthiopyrad (Velista), and (k) propiconazole (Banner Maxx); Figure S2: In vitro growth of *R. solani* isolate Rs_Meyer2019 in fungicide-amended potato dextrose agar (PDA) plate after four days of incubation; (a) control with no fungicide, (b) 100% propiconazole, (c) 100% *B. subtilis* QST713, (d) 25% *B. subtilis* QST713 + 75% propiconazole, (e) 25% *R. sachalinensis* extr. + 75% propiconazole, (f) 50% *B. subtilis* QST713 + 50% propiconazole, (g) 50% *R. sachalinensis* extr. + 50% propiconazole, (h) 75% *R. sachalinensis* extr. + 25% propiconazole, (i) 75% *B. subtilis* QST713 + 25% propiconazole, (j) 50% *B. subtilis* QST713 + 50% *R. sachalinensis* extr., and (k) 100% *R. sachalinensis* extr. Table S1: Bio- and synthetic fungicides, their active ingredients (a.i.), fungicide resistance action committee (FRAC) group, code, product rate, and manufacturer; Table S2: Percent mycelial growth inhibition of *Rhizoctonia solani* isolate RS_Meyer2019 on in vitro assay experiments of treatment alone as well as in combination compared to the non-treated control; Table S3: Disease severity (%) (A) and area under the disease progress curve (AUDPC) (B) for the seven spray programs (T1–T7) across six time points (0–42 days) and two growth chamber experiments carried out at the UGA Griffin Campus, Griffin, GA in 2023; Table S4: Disease severity (%) (A), area under the disease progress curve (AUDPC) (B), and turfgrass quality (1–9) (C) for the seven spray programs (T1–T7) and the seven time points (0–42 days) in the field experiments across both fall 2022 and spring 2023; Table S5: Disease severity (%) (A), area under the disease progress curve (AUDPC) (B), and turfgrass quality (1–9) (C) for three spray programs (T1, T2, and T5) and three time points (0–56 days) in Rivermont Golf Club, Johns Creek, Georgia during spring 2023; Table S6: Analysis of variance of in vitro mycelial growth inhibition of *R. solani* isolate RS_Meyer2019 combined across two experiments (A and D, respectively) as well as under separate analyses (B-C and E-F, respectively); Table S7: Analysis of variance of disease severity (%) (A) and area under the disease progress curve (AUDPC) (B) for seven spray programs against Rhizoctonia large patch across seven time points and two growth chamber experiments carried out at the UGA Griffin Campus, Griffin, GA in 2023; Table S8: Analysis of variance of disease severity (%) (A), area under the disease progress curve (AUDPC) (B), and turfgrass quality (C) for seven spray programs against Rhizoctonia large patch across two seasons and seven time points in the field experiments at the UGA Griffin Campus, Griffin GA; Table S9: Analysis of variance of disease severity (%) (A), area under the disease progress curve (AUDPC) (B), and turfgrass quality (C) for three spray programs against Rhizoctonia large patch and three time points in Rivermont Golf Club, Johns Creek, Georgia during spring 2023; Table S10: Analysis of variance of area under the disease progress curve (AUDPC) for 21 zoysiagrass genotypes against three *R. solani* isolates causing Rhizoctonia large patch under the growth chamber experiment during spring 2023; Table S11: Disease severity for Rhizoctonia large patch on 21 zoysiagrass genotypes for six weeks from 30 to 65 days post-inoculation (dpi) in growth chamber experiment in 2023. Genotypes were categorized into three categories, resistant (R <10% disease severity), intermediate (I = 10–30% disease severity), and susceptible (S > 30% disease severity). Average values with the identical letters across all three isolates within each date are not significantly different according to Tukey’s test (*p* = 0.05); Table S12: Analysis of variance of disease severity (%) for five zoysiagrass breeding lines against *R. solani* isolate Rs_Meyer2019 under the growth chamber experiment in 2022; Table S13: Comparison of disease severity and resistance type across four zoysiagrass gentoypes, including plant introduction PI 231146, that were common to Zuleta et al. (2016) (15 days post-inoculation) and our current study (30 days post-inoculation). Average values with the identical letters within each isolate are not significantly different according to Tukey’s test (*p* = 0.05).
