# Supplementary material for: Assessing Biofungicides and Host Resistance against Rhizoctonia Large Patch in Zoysiagrass
Source: Pathogens. 2024 Oct 2;13(10):864. doi: 10.3390/pathogens13100864 (PMC11510287; doi:10.3390/pathogens13100864)
Supplement: Supplementary file 1 [file pathogens-13-00864-s001.zip › Ghimire et al_MDPI Pathogens_Supp Figure_FINAL.pdf]

Supplementary Figures

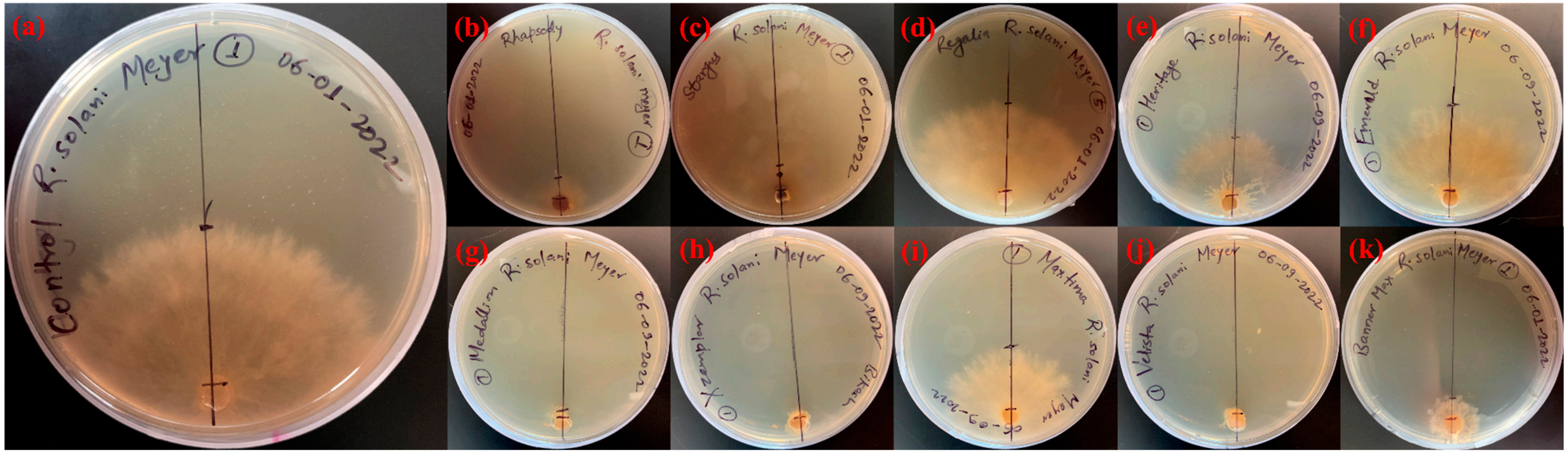

Figure S1: *In vitro* growth of *Rhizoctonia solani* isolate RS_Meyer2019 in fungicide-amended potato dextrose agar (PDA) plate after four days of incubation; (a) control with no fungicide, (b) *Bacillus subtilis* QST713 (Rhapsody), (c) *B. amyloliquefaciens* F727 (Stargus), (d) *Reynoutria sachalinensis* extr. (Regalia), (e) azoxystrobin (Heritage), (f) boscalid (Emerald), (g) fludioxonil (Medallion), (h) fluxapyroxad (Xzemplar), (i) mefentrifluconazole (Maxtima), (j) penthiopyrad (Velista), and (k) propiconazole (Banner Maxx).

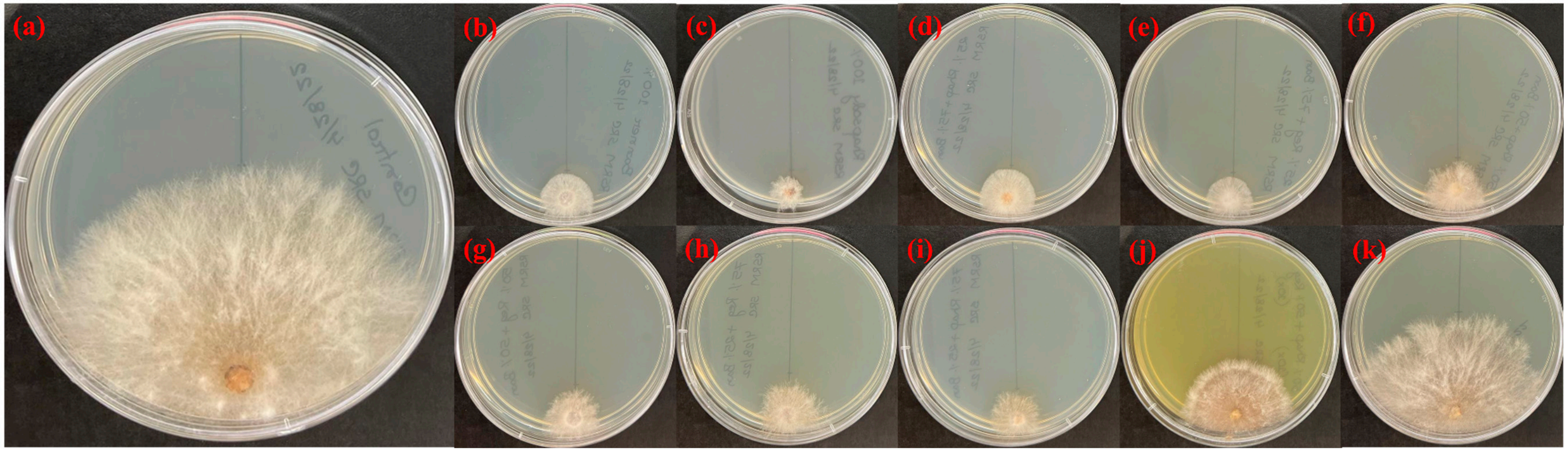

Figure S2: *In vitro* growth of *R. solani* isolate Rs_Meyer2019 in fungicide-amended potato dextrose agar (PDA) plate after four days of incubation; (a) control with no fungicide, (b) 100% propiconazole, (c) 100% *B. subtilis* QST713, (d) 25% *B. subtilis* QST713 + 75% propiconazole, (e) 25% *R. sachalinensis* extr. + 75% propiconazole, (f) 50% *B. subtilis* QST713 + 50% propiconazole, (g) 50% *R. sachalinensis* extr. + 50% propiconazole, (h) 75% *R. sachalinensis* extr. + 25% propiconazole, (i) 75% *B. subtilis* QST713 + 25% propiconazole, (j) 50% *B. subtilis* QST713 + 50% *R. sachalinensis* extr., and (k) 100% *R. sachalinensis* extr.
